# Ethnic disparities in outcomes of coronary artery bypass grafting in Israel

**DOI:** 10.1371/journal.pone.0356423

**Published:** 2026-11-06

**Authors:** Dror B. Leviner, Tom Ronai, Dana Abraham, Ely Erez, Guy Witberg, Yaron Yishai, Joul Haddad, Erez Sharoni

**Affiliations:** 1 Department of Cardiothoracic surgery, Rabin Medical Center, Petach Tikve, Haifa, Israel; 2 Faculty of Medicine, Technion-Israel Institute of Technology, Haifa, Israel; 3 Department of Cardiothoracic surgery, Carmel Medical Center, Israel; 4 Division of Cardiac Surgery, Yale School of Medicine, New Haven, Connecticut, United States of America; 5 Department of Cardiology, Rabin Medical Center, Petach Tikve, Israel; 6 Department of Cardiothoracic Surgery, Soroka Medical Center, Beer Sheba, Israel; 7 Department of Biostatistics, Carmel Medical Center, Haifa, Israel; Baylor Scott and White, Texas A&M College of Medicine, UNITED STATES OF AMERICA

## Abstract

**Objective:**

Ethnic disparities after cardiac surgery are well reported. Israeli Arabs have lower life expectancy and greater frequency of cardiovascular risk factors. We sought to compare long-term outcomes following coronary artery bypass grafting (CABG) between Arab and Jewish patients in Israel.

**Methods:**

Retrospective study of adult patients with multivessel disease undergoing isolated CABG in three centers between January 1^st^, 2009, and December 31^st^, 2023. The primary outcome was a composite of major adverse cardiac and cerebrovascular events (MACCE). The cumulative incidence of MACCE was plotted using KM curves. The hazard ratio (HR) for Arabs vs. Jewish were calculated using multivariate Cox models.

**Results:**

Our cohort included 6,330 patients, 1,819 (28.74%) and 4,511 (71.26%) patients were Arabs and Jewish, respectively. Median follow-up time was 78.32 months. Arab patients had higher rate of the primary outcome at median follow-up (46.26% vs 40.90%, HR 1.184, 95%CI: 1.09–1.286, P < 0.001) and there was no difference in mortality between the two groups (26.44% vs 26.45%, HR 1.004, 95%CI: 0.901–1.118, P = 0.82). After multivariable adjustment, the risk of MACCE was similar between the groups (adjusted HR 1.073, 95%CI: 0.965–1.193, P = 0.19).

**Conclusions:**

After multivariable adjustment for baseline and socioeconomic factors, ethnicity was not independently associated with worse long-term clinical outcomes in patients undergoing CABG in Israel, while unadjusted disparities remained evident.

## Introduction

Coronary artery disease is the most common cause of both heart failure and death worldwide [1, 2], and coronary artery bypass grafting (CABG) is the one of the recommended revascularization options in patients with severe coronary artery disease [3, 4]. Several studies, mainly from the United States (US), have shown higher rates of post-operative mortality and morbidity in black patients undergoing CABG vs. white patients [5–8]. Despite universal health coverage, Arabs in Israel have worse health outcomes and shorter life expectancy than Jewish [9–12]. Moreover, Arab patients with ischemic heart disease (IHD) in Israel have higher mortality rates [13, 14].

Over the last few decades, many studies demonstrated the benefit of second arterial grafting utilization in CABG [15–18]. However, studies have shown that non-white patients were less likely to undergo CABG with multiarterial grafting (MAG) [7, 19].

Hence, the aim of this study was to examine ethnic disparities among patients undergoing isolated CABG in Israel, including utilization of multiarterial grafting, long-term survival, and major adverse cardiac and cerebrovascular events (MACCE).

## Materials and methods

### Design and study population

A retrospective cohort study of patients with multivessel disease undergoing isolated CABG between January 1^st^, 2009, to December 31^st^, 2023, in three cardiac surgery centers in Israel which are affiliated with the same health maintenance organization (HMO). The ethics review board of the three medical centers approved the study in accordance with the Declaration of Helsinki and waived the need for informed consent because of the retrospective nature of the study (Carmel medical center – CMC 21–175, Belinson medical center – RMC – 22–169, Soroka medical center – SOR-22–099).

All adult patients undergoing isolated CABG were included. Patients were excluded if they were members of different HMO (since this results in missing data regarding both comorbidities and limited follow up) and if they underwent any concomitant procedure (i.e., carotid endarterectomy). The patients were divided to two groups according to their ethnicity, Jewish or Arab.

### Configuration and conduits

CABG was performed with conventional technique, either on-pump or off-pump. The attending surgeon in each case chose the conduits and the configuration of the coronary bypass. ITA’s were harvested as skeletonized graft, SVG was harvested either with an open technique or endoscopically, RA was harvested with an open technique.

### Data sources

Data was collected from the electronic medical records of Clalit health services (CHS) – the largest care provider within the Israeli national healthcare system (with 4.9 million members which account for about 54% of the total Israeli population) [20]. CHS maintains a complete and comprehensive electronic medical records which include all medications prescribed and purchased in the community, lab results from both hospitals and the community and International Classification of Diseases, Ninth Revision (ICD-9) coded discharge summaries and billing information. Patient demographic data were collected from the Israeli Central Bureau of Statistics and the Ministry of Internal Affairs, as previously reported [20].

### Outcomes

The primary outcome was a composite of major adverse cardiac and cerebrovascular events (MACCE: all-cause mortality, cerebrovascular accident (CVA), myocardial infarction (MI), and repeat revascularization). Secondary outcomes were each of the components of the primary outcome at long term follow up and outcomes at 30 days after surgery and the utilization of multiarterial grafting.

### Statistical analysis

Continuous variables are summarized with means± standard deviation or median and interquartile range. Categorical variables are presented as numbers and proportions. Comparisons of baseline demographic and clinical characteristics between the two ethnicities were performed using Chi square test for the categorical variables and independent t- test or Mann-Whitney, as appropriate, for the continuous variables.

Kaplan-Meier curves were used to estimate the time to occurrence of any of the MACCE outcomes and compared between Arabs and Jewish using Log rank test.

The annualized incidence rates of MACCE and of each component separately were estimated by dividing the number of incident cases of these outcomes by the total follow-up time and were expressed as number per 100 person-years.

Multivariable Cox proportional hazard model with shared frailties were used to evaluate the association between ethnicity and the different outcomes, accounting for the variability between the three centers. The proportional hazard assumption validated with Schoenfeld Residuals test. In the survival analysis we included the following variables: ethnicity category, socioeconomic status score, age, atrial fibrillation, cancer, heart failure,chronic obstructive pulmonary disease, chronic renal failure,cerebrovascular accident, diabetes, dialysis, hypertension, ischemic heart disease, obesity, peripheral arterial disease, smoking, estimated glomerular filtration rate, MAG, previous percutaneous coronary intervention, ejection fraction range, on/off pump surgery, medical center.

For variables with missing data, an additional category representing missing values was created and included in the analysis, variables with high percentage of missing weren’t included in the analysis. The Interaction between Socioeconomic score and ethnicity is not statistically significant (P = 0.451).

A p-value <0.05 was considered statistically significant. Data analysis was performed using IBM SPSS statistics version 24 (IBM. Ney York, NY), and SAS version 9.4 (SAS institute).

## Results

### Study population

Our initial cohort included 7,067 patients; we removed 737 patients who were members of different HMO. We were left with 6,330 patients who underwent full analysis (Fig 1). Of these, 1,819 (28.74%) were Arabs, and 4,511 (71.26%) were Jewish. Baseline demographics are outlined in Table 1. The 2 groups were relatively low risk and had differences in some of the baseline variables. Arab patients were younger than the Jewish ones (61.21 ± 9.82 vs 65.81 ± 9.42, P < 0.001), had higher rates of diabetes mellitus (65.3% vs 54.7%, P < 0.001), obesity (49.7% vs 41.5%, P < 0.001), smoking (74.3% vs 62.3%, P < 0.001), more Arabs had undergone previous percutaneous coronary intervention (PCI) (39.1% vs 31.4%, P < 0.001) and Arabs underwent off-pump CABG more frequently than Jewish (6.5% vs 4.3%, P < 0.001). There were no differences in left ventricular function nor the utilization of MAG between the two groups.

**Table 1:** Demographic characteristics.

Variable	Arab (N = 1,819)	Jewish (N = 4,511)	P-value	SMD
Age	61.21 ± 9.82	65.81 ± 9.42	**<0.001**	0.478
Male	1,522 (83.7%)	3,705 (82.1%)	0.15	0.041
SES			**<0.001**	2.042
Low	1,738 (95.5%)	1,166 (25.8%)		
Medium	78 (4.3%)	2,848 (63.1%)		
High	0 (0%)	477 (10.6%)		
Diabetes mellitus	1,188 (65.3%)	2,466 (54.7%)	**<0.001**	0.219
COPD	186 (10.2%)	404 (9.0%)	0.12	0.043
Hypertension	1,426 (78.4%)	3,556 (78.8%)	0.72	0.011
Smoker	1,352 (74.3%)	2,811 (62.3%)	**<0.001**	0.260
Dialysis	43 (2.4%)	88 (2.0%)	0.34	0.028
Previous CVA	242 (13.3%)	590 (13.3%)	0.84	0.007
Previous PCI	711 (39.1%)	1,415 (31.4%)	**<0.001**	0.162
Obesity	904 (49.7%)	1,874 (41.5%)	**<0.001**	0.164
PAD	303 (16.7%)	979 (21.7%)	**<0.001**	0.128
eGFR			0.43	0.035
>30	1,722 (94.7%)	4,305 (95.4%)		
<30	72 (4.0%)	153 (3.4%)		
MAG	893 (49.1%)	2,185 (48.4%)	0.65	0.013
Off-Pump	119 (6.5%)	196 (4.3%)	**<0.001**	0.097
LVEF			0.15	0.064
>50%	769 (42.3%)	1,876 (41.6%)		
30-50%	388 (21.3%)	1,024 (22.7%)		
<30%	82 (4.5%)	251 (5.6%)		
Preoperative Hb	13.42 ± 1.81	13.36 ± 1.73	0.21	0.035

Continuous variables are presented with mean ± SD. Discrete variables are reported as number of patients (%). COPD – chronic obstructive pulmonary disease, CVA – cerebrovascular accident, eGFR – estimated glomerular filtration rate, Hb – hemoglobin, LVEF – left ventricular ejection fraction, MAG – multiple arterial grafting, PAD – peripheral artery disease, PCI – percutaneous coronary intervention, SES – socioeconomic status, SMD – standardized mean difference.

Numbers do not sum to 100% due to missing data.

**Fig 1:**
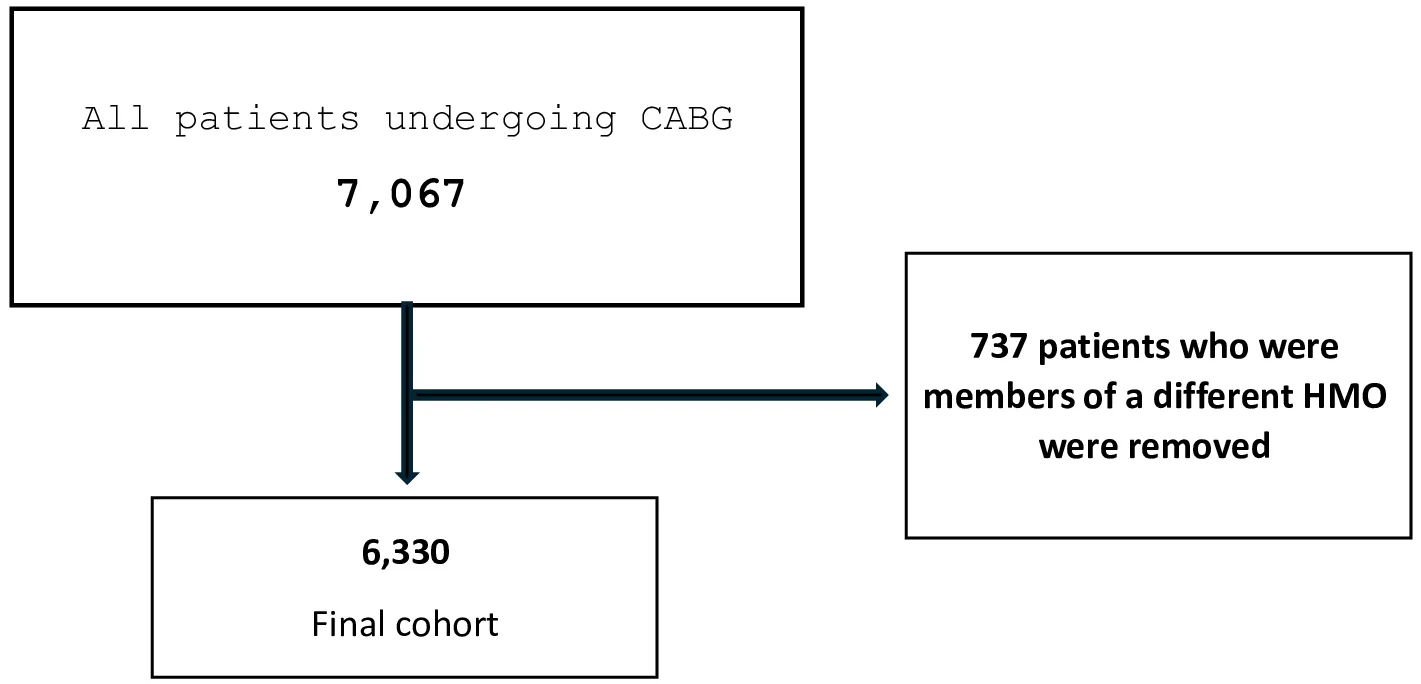
Flow diagram of the patient cohort. CABG – coronary artery bypass grafting, HMO – health maintenance organization.

### In-hospital outcomes

30-day outcomes are outlined in Table 2. 30-day MACCE rates did not differ between the groups (HR 1.02, 95%CI: 0.741–1.289, P = 0.87).

**Table 2:** Post-operative outcomes.

Outcome	Arab (N = 1,819)		Jewish (N = 4,511)		Crude HR	Crude P-value	Adjusted HR	Adjusted P-value
	Events	Incidence rate per 100 years	Events	Incidence rate per 100 years				
30-days								
MACCE	72 (3.96%)	49.5	175 (3.88%)	48.5	1.02 (0.741-1.289)	0.87	1.118 (0.842-1.484)	0.44
PCI	9 (0.5%)		6 (0.1%)					
MI	5 (0.3%)		5 (0.1%)					
CVA	8 (0.4%)		17 (0.4%)					
Mortality	55 (3.0%)		151 (3.3%)					
Long-term								
MACCE	841 (46.26%)	8.1	1,845 (40.90%)	6.9	1.184 (1.09-1.286)	**<0.001**	1.073 (0.965-1.193)	0.19
Revascularization								
PCI	323 (17.73%)	2.9	620 (13.74%)	2.2	1.32 (1.154-1.511)	**<0.001**	1.067 (0.926-1.228)	0.18
CABG	3 (0.16%)		3 (0.07%)					
CVA	171 (9.40%)	1.4	290 (6.43%)	0.9	1.584 (1.306-1.92)	**<0.001**	1.278 (0.997-1.639)	0.052
MI	199 (10.94%)	1.7	345 (7.65%)	1.7	1.468 (1.232-1.748)	**<0.001**	1.187 (0.943-1.493)	0.14
Mortality	481 (26.44%)	3.9	1,193 (26.45%)	3.9	1.004 (0.901-1.118)	0.82	1.055 (0.923-1.205)	0.43

Discrete variables are reported as number of patients (%). CABG – coronary artery bypass grafting, CVA – cerebrovascular accident, HR – hazard ratio, MACCE – major adverse cardiac and cerebrovascular events, MAG – multiple arterial grafting, MI – myocardial infarction, PCI – percutaneous coronary intervention.

### Long-term outcomes

Long-term outcomes are presented in Table 2. Median follow-up in the overall cohort was 78.32 months. Fig 2 outlines the cumulative incidence of MACCE. Cumulative incidence of MACCE was 46.26% among Arab patients and 40.90% among Jewish patients (HR 1.184, 95%CI: 1.09–1.286, P < 0.001). Cumulative incidence of death is presented in Fig 3. At median follow-up, death occurred in similar rates between the groups (26.44% vs 26.45%, HR 1.004, 95%CI: 0.901–1.118, P = 0.82). The incidence of MI (10.94% vs 7.65%, HR 1.468, 95%CI: 1.232–1.748, P < 0.001), stroke (9.40% vs 6.43%, HR 1.584, 95%CI: 1.306–1.92, P < 0.001), and repeat revascularization (17.73% vs 13.74%, HR 1.32, 95%CI: 1.154–1.511, P < 0.001) were significantly higher among Arabs compared to Jewish patients, respectively, at median follow-up.

**Fig 2:**
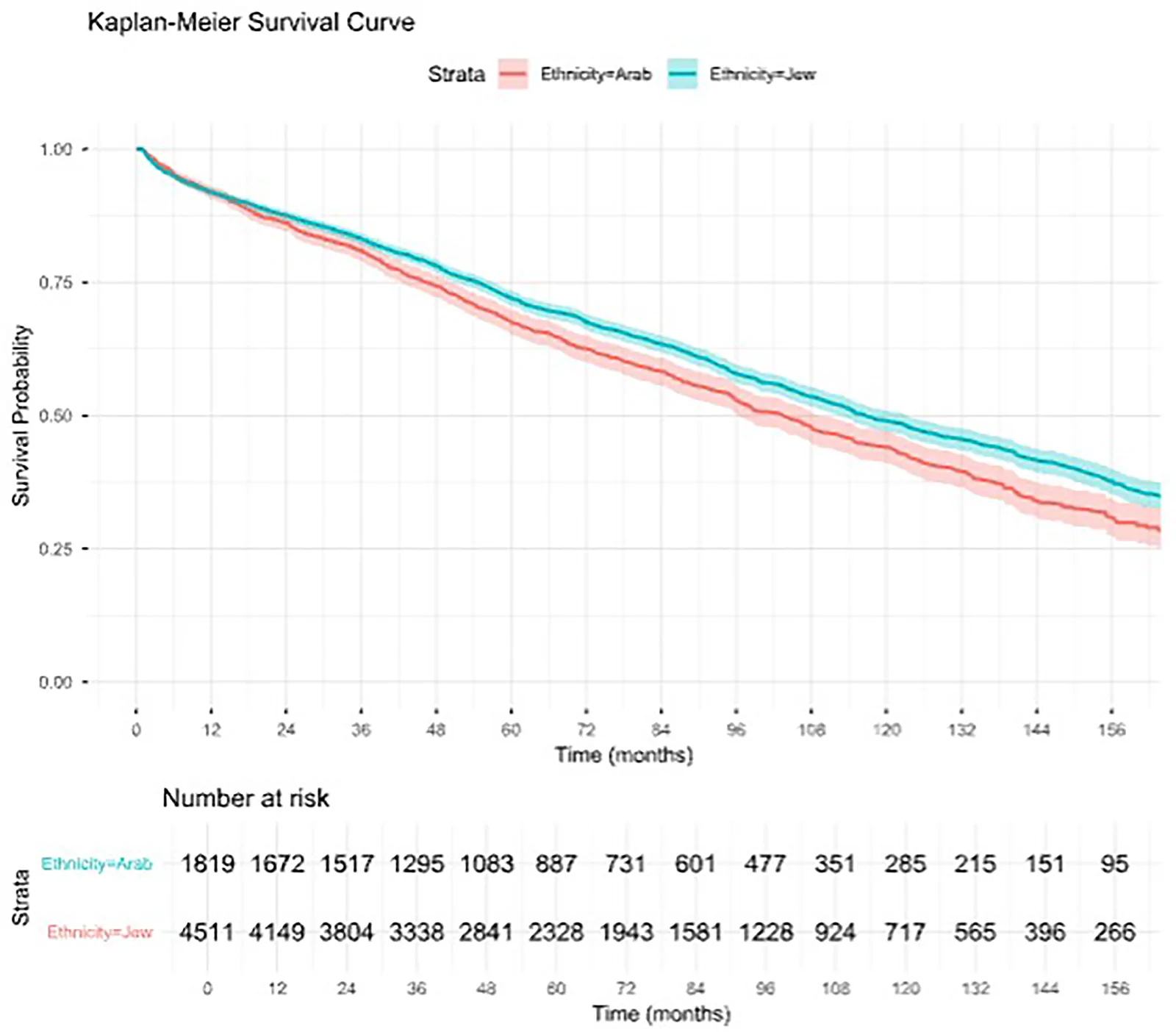
(graphical abstract): Kaplan-Meier curve for MACCE. MACCE – major adverse cardiac and cerebrovascular events.

**Fig 3:**
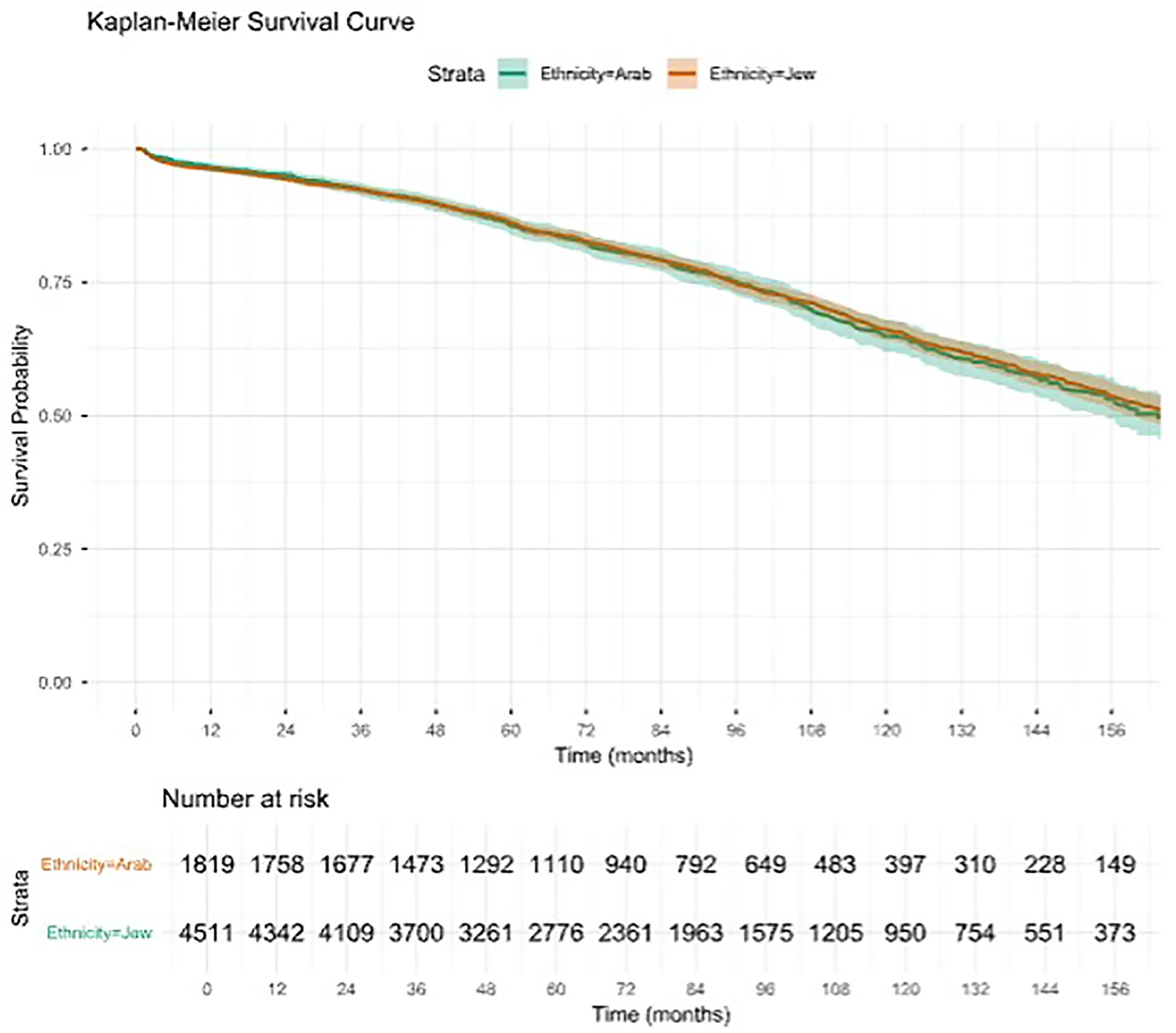
Kaplan-Meier curve for overall mortality.

### Multivariable analysis

After multivariable analysis, there was no significant difference in the risk of MACCE (adjusted HR 1.073, 95%CI: 0.965–1.193, P = 0.19) or mortality (adjusted HR 1.055, 95%CI: 0.923–1.205, P = 0.43) at median follow-up between the groups. The risk of MI (adjusted HR 1.187, 95%CI: 0.943–1.493, P = 0.14), stroke (adjusted HR 1.278, 95%CI: 0.997–1.639, P = 0.052) and repeat revascularization (adjusted HR 1.067, 95%CI: 0.926–1.228, P = 0.18) were similar between the two groups.

### Comment

In this analysis, we assessed for ethnic differences in Israeli patients undergoing isolated CABG. We found several important findings of note. First, Arab patients had higher rate of MACCE than Jewish, but after multivariable adjustment, the risk of MACCE was similar between the groups over a median follow-up of 78 months. Second, there were no differences regarding conduit choice between the groups.

There are several possible explanations for our findings. First, Arab patients underwent CABG at a younger age, with a higher proportion of comorbidities (i.e., hypertension, diabetes) and a lower socioeconomic status than Jewish. The higher rate of comorbidities could account for some of the difference we found in the incidence of MACCE among Arab patients. Several studies have shown that even though there is a universal health system in Israel, Arab patients have worse clinical outcomes and shorter life expectancy than Jewish patients [9–11]. Abu-Much *et al.* conducted a long-term follow-up of patients with stable IHD and used PSM to analyze ethnic disparities. They demonstrated that Arab patients with IHD had higher 20-year all-cause mortality compared to Jewish and multivariable analysis showed a greater mortality risk among Arabs [14]. Moreover, Karkabi *et al.* showed that Arab patients had lower 1-year survival after acute coronary syndrome compared to Jewish patients [13]. Second, studies have shown that Arab patients make less frequent use of ambulatory health services after MI and are less likely to be prescribed advanced medical therapy [21–23]. Reges *et al.* showed that 6 months following acute coronary syndrome, Arab patients were less likely to cease smoking, to conduct regular physical exercise and attend cardiac rehabilitation programs [24]. The importance of secondary prevention after CABG is well established. First, adherence to pharmacological therapy is crucial for preventing recurrent MI and decreasing mortality [25, 26]. Second, lifestyle modification including smoking cessation, regular physical activity and participation in cardiac rehabilitation programs has been shown to confer a survival benefit [27]. Thus, these differences in long-term treatment most likely impact the incidence of MACCE.

Ethnic and racial disparities in cardiac surgery have been demonstrated in several large studies that showed higher mortality and morbidity among black patients compared to white patients [5, 6, 28, 29]. Specifically, black patients who underwent CABG are at higher risk for both in-hospital and long-term mortality. Enumah *et al.* performed a retrospective analysis of 1 million patients from the Society of Thoracic Surgery (STS) database of patients undergoing CABG and showed higher 30-days mortality rate of black patients compared to white patients [5]. Benedetto *et al.* conducted a risk-adjusted meta-analysis of racial differences regarding in-hospital mortality of patients undergoing CABG and showed that despite progress over the last few years, black patients are still at higher risk for in-hospital mortality compared to white patients [6]. Zea-Vera *et al.* conducted a national analysis of more than 2 million patients who underwent CABG in the US and after adjustment for perioperative risk factors concluded that black patients had a higher mortality rate following CABG compared to white patients [8].

Finally, after multivariable adjustment for baseline differences between the groups, the risk of MACCE was similar between the groups. Multiple studies have demonstrated the benefit of a second arterial conduit on both mortality and MACCE [15–18, 30]. However, around 85% of patients undergoing CABG are still receiving only one arterial conduit [7, 31, 32]. Prior analyses showed that black patients in the US are less likely to undergo CABG with MAG. Rotar *et al.* examined whether ethnic and racial disparities exist in practice during CABG and showed that white patients more often received MAG and were more likely to receive complete revascularization compared to black patients [7]. Tabata *et al.* performed a retrospective analysis of half a million patients from the STS database who underwent CABG and also concluded that non-white patients received MAG less often than white patients [19]. Moreover, Trivedi *et al.* showed in their retrospective analysis of more than 700,000 patients in the US that black patients were more likely to undergo CABG at low-volume center and that there is a significant interaction between the volume-center and mortality rate following CABG [33]. Rangrass *et al.* conducted a retrospective analysis of more than 150,000 patients who underwent CABG in the United Sates and concluded that hospital quality accounted for approximately 35% of the ethnic disparity in mortality after CABG in blacks compared to white patients [34]. More recently, Popescu *et al.* also showed that white patients were more likely to undergo CABG at high-quality hospitals compared to black patients and that these differences were not explained by geographic factors alone [35]. In our analysis, the utilization of MAG was common and similar between the groups, and the 2 groups underwent surgery at the same 3 centers. Thus, it seems that when operated on in the same hospitals, ethnic minorities are likely to receive similar quality care regardless of socioeconomic status and ethnic differences.

### Limitations

This is a retrospective, non-randomized cohort with all the inherent limitations, however, multivariable analysis was utilized to compensate for the differences in the demographic background of the 2 groups. However, to our understanding there is no other method to compare 2 different ethnic groups. Unfortunately, data regarding the extent of the coronary artery disease before surgery (i.e., SYNTAX score, left main disease) and data regarding the operative strategy (i.e., conduits selection, sequential grafting, number of distal anastomoses, completeness of revascularization, target vessel quality) were not available for analysis.

## Conclusion

In this large cohort of patients undergoing isolated CABG, after multivariable adjustment for baseline and socioeconomic factors, there were no difference in postoperative outcomes or with different utilization of MAG, while unadjusted disparities remained evident. The disparities between the two groups are most likely related to baseline risk factors and socioeconomic determinants. Moreover, the similar rates of MAG usage in the two groups imply that to improve long-term post-operative results in the Arab minority in the current setting, the focus should be on long term care and improvement of access to healthcare.

## Abbreviations

CABG: coronary artery bypass grafting
MACCE: major adverse cardiac and cerebrovascular events
MAG: multiarterial grafting
